# Impact of aortocaval shunt flow on cardiac and renal function in unilateral nephrectomized rats

**DOI:** 10.1038/srep27493

**Published:** 2016-06-09

**Authors:** Jie Wu, Zhong Cheng, Mingjing Zhang, Pengfei Zhu, Ye Gu

**Affiliations:** 1 Department of Cardiology, Puai Hospital, Jianghan University, Wuhan, 430033, China

## Abstract

We previously reported significantly enhanced cardiac remodeling post aortocaval fistula (AV) in unilateral nephrectomized (UNX) rats. However, the relationship between the size of the AV and the cardiorenal effects in UNX rats remains unknown. In the present study, AV was induced by 20, 18 and 16 gauge needles in UNX rats to see if larger shunt would definitely induce heavier cardiac and renal damage in UNX rats. Our results demonstrated that bigger shunt size is linked with proportional more significant cardiorenal remodeling and dysfunction in UNX rats. Expression of inflammatory biomarkers including CRP, TNF-α, IL-6, IL-1β, TGF-β and MCP-1 in left kidney and heart was significantly increased in all UNX + AV groups compared to Sham rats. Inflammation might thus participate in the worsening cardiorenal functions and remodeling processes in this model.

Previously, we reported that cardiac remodeling post aortocaval fistula (AV) was significantly enhanced in unilateral nephrectomized (UNX) rats compared to rats without UNX^1^. According to our results, healthy living donors might face increased cardiac and renal dysfunction risk in case of future chronic volume overload insult, as in the case of pregnancy and other high output heart failure situations as in the case of hyperthyroidism, anemia and in patients with arteriovenous fistula^1^. However, the relationship between different degrees of volume overload and adverse cardiorenal remodeling in UNX rats is still not fully understood. In the previous report, AV was established by 18-gauge needle puncture. In the present study, we performed new experiments by using 20, 18 and 16 gauge needles to induce AV in UNX rats. The main objective was to explore if larger shunt would definitely induce heavier cardiac/renal dysfunction in UNX rats.

## Results

### Survival and general characteristics

Three rats died during the 8 weeks post operation, one died on the first day post AV in group AV18 (AV produced by 18 gauge needle) and post mortem examination revealed massive bleeding around the puncture site, and the death might thus be caused by failure of sealing the puncture site by cyanoacrylate; one died on the 14^th^ day post operation in group AV20 (AV produced by 20 gauge needle) and another died on the 12^th^ day post operation in group AV16 (AV produced by 16 gauge needle). Post mortem examination visualized hypertrophied heart, congested liver and lung, presence of ascites and pleural effusion, as well as edema of the limbs, suggesting that overt congestive heart failure might be the cause of death in these rats died around two weeks after AV operation. Food and water intake did not differ between the groups, and ascites and pleural effusion were not evidenced in the rats survived to study end. Compared to the UNX alone group of previous study^1^, UNX + AV increased perioperative mortality (8% vs. 0%).

### Body weight and organ weights, morphological changes

**Figure 1:**
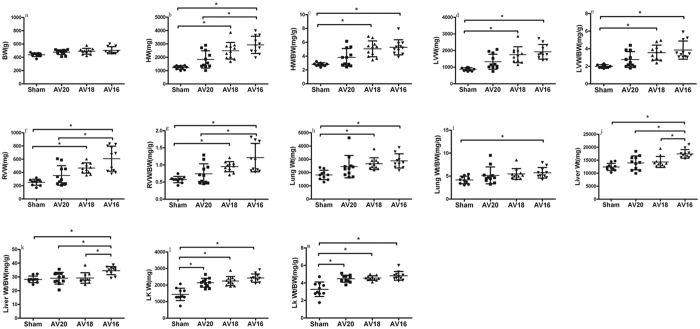
Changes in body weight and organ weights in response to three different shunt size in UNX + AV rats. Values are mean ± SD. BW, Body weight (**a**); HW, Heart weight (**b**); HW/BW (**c**); LVW, left ventricular weight (**d**); LVW/BW (**e**); RVW, right ventricular weight (**f**); RVW/BW (**g**); Lung Wt, Lung wet weight (**h**); Lung Wt/BW (**i**); Liver Wt (**j**); Liver Wt/BW (**k**); LK Wt, left kidney Wt (**l**); LK Wt/BW (**m**). *p < 0.05.

Body weight and organ weights are shown in Fig. 1. Eight weeks post various procedures, body weight was similar among groups. Heart weight and heart/body weight ratio, LVW (mg), LVW/BW (mg/g), RVW (mg) and RVW/BW (mg/g) in group AV20, AV18 and AV16 tended to be higher than in Sham group with the highest value in group AV16. Wet lung weight and wet lung/BW ratio, liver wet weight and liver wet weight/BW ratio, left kidney wet weight and left kidney weight/BW also tended to be higher in group AV20, AV18 and AV16 compared to Sham group and the highest value was still seen in group AV16. Compared to the UNX alone group from the previous study^1^, the left kidney wet weight/body weight ratio (4.31 ± 1.63 mg/g in UNX) was similar while heart weight/body weight ratio (2.56 ± 0.17 mg/g in UNX), left ventricular weight/body weight ratio (1.93 ± 0.11 mg/g in UNX), right ventricular weight/body weight ratio (0.45 ± 0.07 mg/g in UNX) and lung wet weight/body weight ratio (3.34 ± 0.44 mg/g in UNX) tended to be higher in all UNX + AV groups with the highest value in group AV16. The liver wet weight/body weight ratio was similar among AV20, AV18 and UNX alone (28.96 ± 3.81 mg/g) groups in the previous study^1^ while significantly increased in AV16 compared UNX alone group in the previous study^1^.

### Echocardiography measurements

As shown in Table 1, LVEDD and LVESD tended to be higher while LVEF and LVFS values tended to be lower in group AV18 and AV16 than in Sham group and group AV20. There is a trend of more severe LV remodeling and dysfunction with the increased fistula sizes. Compared to the UNX alone group from the previous study^1^, LVEDD (5.960 ± 0.659 mm in UNX) and LVESD (3.162 ± 0.686 mm in UNX) tended to be higher while LVEF (86.44 ± 4.22% in UNX) and LVFS (50.78 ± 5.74% in UNX) values tended to be lower in all UNX + AV groups.

### Renal perfusion pressure

As shown in Table 2, The UNX + AV operation decreased the mean renal artery pressure and renal perfusion pressure and increased the renal vein pressure. These changes were more significant in proportion to larger fistula size. Compared to the UNX alone group from the previous study^1^, the mean renal artery pressure (126.4 ± 15.4 mmHg in UNX) was lower and the renal vein pressure (5.1 ± 0.9 mmHg in UNX) was higher in all UNX + AV groups. These changes were more apparent in AV16 group.

### Biochemistry and renal function parameters

**Figure 2:**
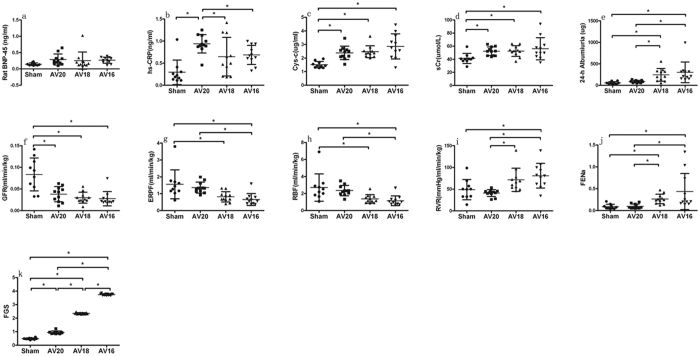
Changes in biochemistry and renal function parameters in response to three different shunt size in UNX + AV rats. Values are mean ± SD. BNP-45, rat brain natriuretic peptide-45 (**a**); hs-CRP, hypersensitive C-reactive protein (**b**); Cys-c, Cystatin C (**c**); sCr, plasma creatinine (**d**); 24 h Albumiuria (**e**); GFR, glomerular filtration rate (**f**); ERPF, effective renal plasma flow (**g**); RBF, renal blood flow (**h**); RVR, renal vascular resistance (**i**); FENa, fractional excretion of sodium (**j**); FGS, focal segmental glomerulosclerosis quantification (**k**). *p < 0.05.

As shown in Fig. 2, rat brain natriuretic peptide-45 tended to be slightly higher post UNX + AV operations compared to Sham group, but the value was similar among group AV20, AV18 and AV16. Plasma Hs-CRP levels in group AV20, AV18 and AV16 tended to be higher than in Sham group, with the highest value in group AV20. Plasma creatinine and cystatin C levels in group AV20, AV18 and AV16 also tended to be higher than in Sham group, with the highest value in group AV16. GFR was significantly and equally reduced in group AV18 and AV16 compared to Sham group, ERPF and RBF were significantly decreased while RVR and FENa were significantly increased in group AV20, AV18 and AV16 compared to Sham group. These changes were more significant in proportion to larger fistula sizes. 24 hours albuminuria was higher in group AV18 and AV16 than in Sham and AV20 group. Focal segmental glomerulosclerosis quantification showed FGS was significantly increased in group AV20, AV18 and AV16, and the highest FGS value was seen in group AV16. Compared to the UNX alone group from the previous study^1^, rat brain natriuretic (0.17 ± 0.03 ng/ml in UNX) tended to be higher in all UNX + AV groups and there was a trend for more apparent changes in AV16 group. In proportion to AV size, especially in group AV18 and AV16, GFR (0.045 ± 0.015 ml/min/kg in UNX), ERPF (0.871 ± 0.712 ml/min/kg in UNX), RBF (1.468 ± 1.200 ml/min/kg in UNX) further decreased and FENa (0.120 ± 0.075 in UNX), 24 h Albumiuria (113.35 ± 127.44 ug in UNX) and focal segmental glomerulosclerosis quantification (0.620 ± 0.030 in UNX) further increased compared to the UNX alone group from the previous study^1^.

### Inflammatory biomarkers mRNA expression in kidney and heart

**Figure 3:**
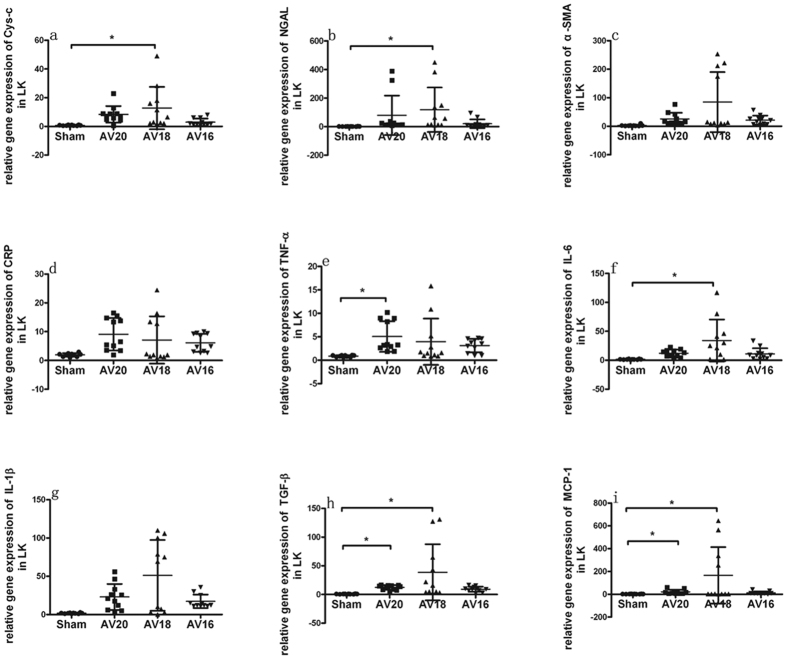
Changes in mRNA expression in left kidney in response to three different shunt size in UNX + AV rats. Values are mean ± SD. Cys-c, Cystatin C (**a**); NGAL, neutrophil gelatinaseassociated lipocalin (**b**); α-SMA, α-smooth muscle actin (**c**); CRP, C-reaction protein (**d**); TNF-α, tumor necrosis factor-α (**e**); IL-6, interleukin-6 (**f**); IL-1β, interleukin-1β (**g**); TGF-β, transforming growth factor-β (**h**); MCP-1, monocyte chemotactic protein 1 (**i**). *p < 0.05.

**Figure 4:**
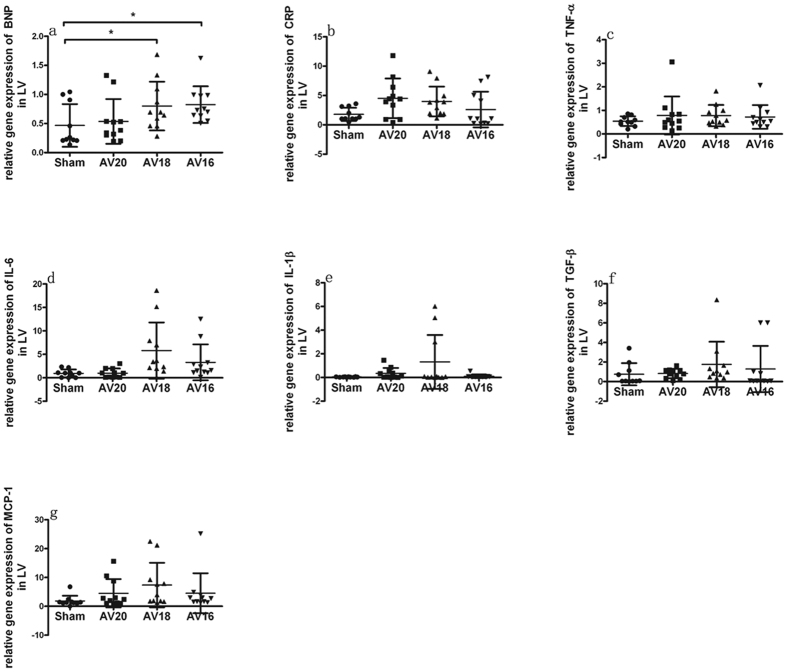
Changes in mRNA expression in left ventricle in response to three different shunt size in UNX + AV rats. Values are mean ± SD. BNP, brain natriuretic peptide (**a**); CRP, C-reaction protein (**b**); TNF-α, tumor necrosis factor-α (**c**); IL-6, interleukin-6 (**d**); IL-1β, interleukin-1β (**e**); TGF-β, transforming growth factor-β (**f**); MCP-1, monocyte chemotactic protein 1 (**g**). *p < 0.05.

**Figure 5:**
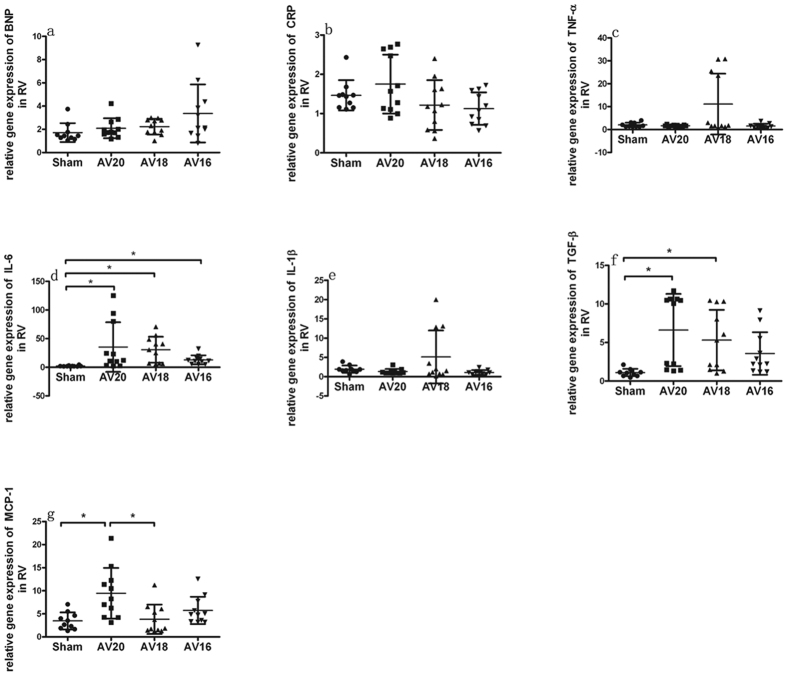
Changes in mRNA expression in right ventricle in response to three different shunt size in UNX + AV rats. Values are mean ± SD. BNP, brain natriuretic peptide (**a**); CRP, C-reaction protein (**b**); TNF-α, tumor necrosis factor-α (**c**); IL-6, interleukin-6 (**d**); IL-1β, interleukin-1β (**e**); TGF-β, transforming growth factor-β (**f**); MCP-1, monocyte chemotactic protein 1 (**g**). *p < 0.05.

As shown in Fig. 3, the kidney injury biomarkers such as Cys-c, NGAL and α-SMA expressions in left kidney tended to be higher in group AV20, AV18 and AV16 compared with Sham group. The highest expression was seen in group AV18. Inflammation biomarkers such as IL-6, IL-1β, TGF-β and MCP-1 expressions in left kidney also tended to be higher in group AV20, AV18 and AV16 compared with Sham group and the highest expression was seen in group AV18. CRP and TNF-α expression tended to be higher in group AV20, AV18 and AV16 compared to Sham group. As shown in Figs 4 and 5, mRNA expression of BNP, CRP, TNF-α, IL-6, IL-1β, TGF-β and MCP-1 in left and right ventricle also tended to be higher in group AV20, AV18 and AV16 compared to Sham group. For most parameters in left ventricle, the highest expression was seen in AV18 group. On the other hand, for most parameters in right ventricle, the highest expression was seen in AV20 group.

## Discussion

As described in our previous report, although UNX only induces minor renal dysfunction, additional chronic volume overload placement during the adaptation phase of the remaining kidney is associated with aggravated cardiorenal dysfunction and remodeling in UNX rats^1^. In 2012, Martin *et al.* reported that UNX induced mild CKD could result in early cardiac fibrosis in case of mild diastolic impairment and preserved systolic function, the cardiac hypertrophy and impairment of heart function progressed over time^2^. This kidney-heart interaction seems to be independent of increase in blood pressure, sodium, or water retention, or activation of aldosterone^2^. In this study, we observed if the shunt volume would be a critical determinant for above changes, i.e. the larger the shunt, the bigger the harm. So we used the previous experimental settings and induced various AV shunt flow by using 3 needle sizes. The major findings of present study are as follows: (1) LV dilation is proportional to AV shunt flow. The extent of hypertrophy was greater in RV than that of the LV. (2) Renal function was significantly deteriorated in this model in proportion to AV flow. (3) Above changes are concomitant with upregulated mRNA expression of inflammatory and tissue injury biomarkers in the left kidney and heart.

In our model, the shunt size was controlled by using different sizes of needles. As mentioned in previous studies^3^, the differently sized fistulas were useful in creating different degrees of congestive heart failure. This is exemplified by a distinct difference between the remodeling and functional parameters seen in the AV groups produced using the 20, 18 and 16 gauge needle in this study. Rats in AV16 group underwent transition to decompensated heart failure (reflected by the significantly greater right ventricular weight, LV dilatation, increased lung and liver weight), whereas rats in AV18 group were still in the compensatory phase of remodeling and rats in AV20 group only showed signs of myocardial and renal hypertrophy. The criteria for making above determinations has been well characterized in the previous aortocaval fistula induced heart failure rat models by several authors^4^,^5^. Some researchers have pointed out the hemodynamic challenges to the LV and RV post AV are different. RV faced both volume and pressure overload challenges, thus exhibited a more rapid and greater hypertrophic response^6^, in fact, proportional RV hypertrophy was observed with increasing AV shunt flow (Fig. 1). For the LV, there is a trend of aggravation in cardiac remodeling and dysfunction with increasing AV shunt flow (Fig. 1), the reason for absence of significant differences between the three shunt-groups (AV20, AV18 and AV16) could be that we performed the AV at 1 week post UNX, the time interval between the two operations as well as the observation time might be too short to validate the cardiac performance differences in the LV. So, future studies with various experimental settings are required to determine the impacts of various gauge size, the various timing of AV operation post UNX surgery and post AV observation period of this model on cardiac and renal outcome as well as the effects of various pharmaceutical interventions in this model.

We previously showed, UNX alone induced a statistically significant compensatory hypertrophy of the remaining kidney while the renal dysfunction was very mild^1^. Results from present study demonstrate that glomerulosclerosis worsened, proteinuria progressed, ERPF, RBF further decreased and RVR, FENa, plasma creatinine and cystatin C level further increased while the kidney injury biomarkers such as Cys-c, NGAL and α-SMA expressions in left kidney tended to be higher in proportion to AV size. Thus, renal remodeling and dysfunction are shunt flow-dependent processes in this model, suggesting the hemodynamic factor may be the most important determinant in AV induced renal remodeling and dysfunction. The two important hemodynamic variables that linking the heart and kidneys are renal blood flow and renal perfusion pressure (RPP). RPP was usually defined as mean arterial pressure minus renal venous pressure^7^. Traditionally, cardiorenal syndrome (CRS) has been interpreted as a consequence of an insufficient renal perfusion^7^,^8^, as a result of “forward” heart failure. Recently, there was renewed interest with the concept of a backward transmission of central venous pressure (CVP) elevation leading to renal dysfunction^7^, a scenario of “backward” heart failure^9^,^10^,^11^. In our study, creation of AV fistula results in an immediate and sustained decrease in mean arterial pressure together with a substantial increase in venous blood flow to the right heart as evidenced by increased RVP. The increase in pressure in the venous system in rats with larger fistula shunt flow should be higher than that in rats with smaller fistula shunt flow. Although an increase in RVP does not always correspond to increased venous blood flow, but rather results in congestive heart failure, as a consequence of increased back flow or stasis in the venous return. As shown in Table 2, the RPP value was lowered to ≤80 mm Hg in AV16 group, that is the threshold of kidney autoregulation, which could lead to the decrease in renal perfusion, and the latter might be responsible for the further decrease in glomerular filtration rate in AV rats with larger shunt flow. It is worth noting that the blood pressure was reported to remain unchanged or only slightly lower in rat AV model^12^, observed reductions in renal hemodynamics in our study might possibly an artifact of anesthesia. This should be acknowledged as a potential limitation.

In addition to the traditional intra- and extra- renal hemodynamics effects post AV, there has been a growing interest in inflammatory and endothelial cell activation in the AV-induced heart failure pathogenesis. It has been proposed that in case of heart failure, the endothelium changes from a quiescent redox profile to an activated proinflammatory, prooxidant, and provasoconstrictive state^13^. It was theorized that venous congestion might cause a biochemical signal to endothelial cells inducing changes on redox phenotype of reactive oxygen species, endothelin, interleukin-6, tumor necrosis factor alpha, and nitric oxide bioavailability^14^. Thus, volume overload and venous congestion could serve as a source of inflammatory mediators. These biomarkers activated as part of the inflammatory process and might serve as major contributors to renal and heart hypertrophy/fibrosis. In our study, expression of biomarker like TGF-β, CRP, TNF-α, IL-6, IL-1β and MCP-1 in left kidney and heart all tended to be higher in UNX + AV groups compared to Sham group, thus suggesting a potential role of these cytokines in modulating the cardiac and renal remodeling in this model. It is to note, the highest levels of these cytokines were not seen in AV16 group. In left ventricle, prominent changes were seen in AV18 group. In right ventricle, prominent changes were seen in AV20 group. The underlying reason remains unclear now. It could be speculated that the dynamic inflammatory changes might differ upon various degree of AV flow in this model. The reliance on mRNA may not reflect the actual levels of the cytokines in kidney and heart. Future studies with a more quantitative assessment of protein changes by Western blot analysis, as well as better characterization of the remodeling are needed to address the specific causal or mechanistic events underlying the observed differences.

Taken together, our results show that AV induced cardiac and renal remodeling in UNX rats are AV flow dependent and inflammatory cytokines might play potential role in volume overload induced cardiac and renal remodeling in this model.

## Materials and Methods

### Experimental Animals

Study protocol and experiments were approved by the Jianghan Medical College Council on the Animal Care Committee of Jianghan University (Wuhan, China). Animals were maintained in accordance with the Guide for the Care and Use of Laboratory Animals published by the US National Institute of Health (NIH Publication No. 85-23, revised 1996). All surgery was performed under sodium pentobarbital anesthesia, and all efforts were made to minimize suffering as described previously^1^. Male Sprague-Dawley (SD) rats (weighing 200 to 250 g) were housed under standard conditions with free access to food and drinking water. Rats received a normal salt diet (0.3% NaCl) throughout the study.

### Overall study setup and study groups

Rats were randomly divided into Sham (n = 10), AV20 (AV by 20-gauge angiocath at one week after UNX, n = 12), AV18 (AV by 18-gauge angiocath at one week after UNX, n = 12) and AV16 (AV by 16-gauge angiocath at one week after UNX, n = 12), respectively. We omit the nephrectomy only group, since the changes post nephrectomy were already described previously^1^ and obtained results were compared with data in nephrectomy only rats from the previous study^1^. At time (t0) = 0 wk , all rats were subjected to UNX or sham operation. At time (t1) = 1 wk, rats were subjected to AV (AV established between the levels of renal arteries and iliac bifurcation) by 20−, 18−, and 16-gauge angiocath needles or sham operation. Rats were followed up to week 9. After eight weeks, survived rats were placed in individual metabolic cages and two consecutive 24-hour urine samples were collected after 5 adaptation days. Echocardiography was performed two days after the metabolic cage studies. Invasive hemodynamic and renal function measurements were applied to rats after echocardiography examination. Blood sample was then collected from the vena cava. Finally, rats were sacrificed under deep anesthesia (70 mg/kg sodium pentobarbital intraperitoneally), and organs were removed, weighed, and processed for histological and molecular examinations.

### Surgical Procedures

UNX operation was performed as previously described. Briefly, the right kidney was carefully separated from the adrenal gland and the surrounding tissue^15^, then removed after laparotomy under anesthesia with 1% pentobarbital sodium salt (40 mg/kg intraperitoneal injection). AV was established on UNX rats at 1 week post right kidney removal with the method described by Garcia and Diebold^16^ with 20− or 18− or 16-gauge angiocath needles, respectively. Sham rats underwent similar surgical procedures as rats in UNX + AV group, but right kidney was not removed and AV was not established in sham rats.

### Echocardiography Examination

Echocardiography examination was performed by an investigator blinded to the study protocol with an echocardiographic system (GE Vivid 7) equipped with a 11.4 MHz transducer as previously described^1^. Left ventricular end-diastolic diameter (LVEDd), left ventricular end-systolic diameter (LVEDs), left ventricular ejection fraction (LVEF) and left ventricular fractional shortening (FS) were determined. Mean values of at least three consecutive cardiac cycles were used^17^.

### Hemodynamic studies *in vivo*

Two days post echocardiography examination, left and right heart catheterization was made in pentobarbital sodium salt anesthetized (40 mg/kg, intraperitoneal injection) rats as previously described^1^. The mean renal artery pressure (RAP) and renal vein pressure were measured according the method described by Dong *et al*^18^ and the renal perfusion pressure (RPP) was defined as RAP minus RVP.

### Renal function measurements

Renal function measurements were made as previously described^1^. Briefly, the jugular vein was cannulated, intravenous inulin/para-aminohippurate (PAH) solution was injected as a bolus (2 ml/kg), then continuously via an infusion pump (25 μl/min). One hour later, hemodynamics and renal function were determined for 90 min (three 30 min clearance periods). Urine was collected at 30 min intervals through the bladder catheter, arterial blood samples (500 μl) were collected via the carotid artery catheter at the midpoint of each urine collection period into prechilled heparinized tubes for related measurements.

### Histology

After blood sampling from vena cava, the rats were sacrificed under additional deep anesthesia (70 mg/kg sodium pentobarbital intraperitoneally). The heart, left kidney, lung and liver were removed and immediately placed in ice-cold saline to wash out the blood. Total heart, right ventricular (RV), left ventricular (LV) weight, left kidney wet weight, lung wet weight and liver wet weight were measured after the removal of connective tissue; the septum was included in the LV. One part of the kidney was fixed in 40 g/l formaldehyde, embedded in paraffin, cut into sections and stained by hematoxylin and eosin (H&E) and periodic acid-Schiff (PAS) for light microscopy. The incidence of focal glomerulosclerosis (FGS) was determined as previously descried^19^. Gross glomerulosclerosis score was calculated as a summation of . The remaining kidney and heart tissues were snap-frozen in liquid nitrogen.

### Clinical Chemistry

24-hour urine albumin, plasma creatinine, plasma rBNP-45 concentrations, plasma cystatin C concentrations, plasma hs-CRP concentrations, inulin, plasma and urine PAH concentrations, GFR, clearance of inulin were determined as previously described^1^. Effective renal plasma flow (ERPF), renal blood flow, renal vascular resistance and fractional excretion of sodium was calculated as previously described in detail^1^.

### Real-time polymerase chain reaction measurements

Total RNA was extracted from renal cortical tissue and left/right ventricular free wall using Total RNA kit (Takara, Japan) according to the manufacturer’s instructions. Reverse transcription and cDNA synthesis were accomplished using RNA PCR kit (Takara, Japan). Real-time polymerase chain reaction was performed to detect the expression of various cytokines by Step One SYBR Green Mix Kit (Takara, Japan) and ABI Prism Sequence Detection System (Applied Biosystems, USA) according to the manufacturer’s instructions. The conditions of amplification reaction were 95 °C for 30 sec, 95 °C for 5 sec, 60 °C for 30 sec, and PCR was done for 40 cycles. PCR primers are shown in Table 3. Relative gene expression was calculated using the 2^−ΔΔCT^ method.

### Statistical Analyses

All data are presented as mean ± SD. Differences between groups in mean values with normal distribution were compared by one-way ANOVA followed by Tukey test, otherwise a kruskal-wallis test followed by Mann-Whitney U test with Bonferroni correction was used. P < 0.05 was considered statistically significant.

## Additional Information

**How to cite this article**: Wu, J. *et al.* Impact of aortocaval shunt flow on cardiac and renal function in unilateral nephrectomized rats. *Sci. Rep.* **6**, 27493; doi: 10.1038/srep27493 (2016).

## Figures and Tables

**Table 1:** Echocardiographic Parameters.

	Sham(n = 10)	AV20(n = 11)	AV18(n = 11)	AV16(n = 11)
LVEDD(mm)	6.418 ± 0.305	6.296 ± 0.516	7.618 ± 0.41	8.85 ± 1.13*†
LVESD(mm)	3.333 ± 0.396	3.334 ± 0.576	4.404 ± 0.576	4.988 ± 1.165*†
EF(%)	84.33 ± 4.50	83.40 ± 5.32	81.20 ± 2.39	79.38 ± 7.05
FS(%)	48.00 ± 5.55	47.00 ± 6.04	44.40 ± 1.34	44.25 ± 7.13

Values are mean ± SD. LVEDD, left ventricular end-diastolic dimension; LVESD, left ventricular end-systolic dimension; EF, ejection fraction; FS, fractional shortening. *p < 0.05 vs. Sham; ^†^p < 0.05 vs. AV20; ^‡^p < 0.05 vs. AV18.

**Table 2:** The parameters of renal hemodynamics.

	Sham(n = 10)	AV20(n = 11)	AV18(n = 11)	AV16(n = 11)
MRAP(mmHg)	104.3 ± 9.4	92.8 ± 1.4	87.8 ± 9.8*	79.9 ± 7.5*†
RVP(mmHg)	3.1 ± 1.3	5.9 ± 0.9*	8.9 ± 1.8*†	11.4 ± 2.1*†‡
RPP(mmHg)	101.2 ± 3.3	86.9 ± 1.1	78.9 ± 1.8*	68.5 ± 8.7*†

Values are mean ± SD. MRAP, mean renal artery pressure; RVP, renal vein pressure; RPP, renal perfusion pressure. *p < 0.05 vs. Sham; ^†^p < 0.05 vs. AV20; ^‡^p < 0.05 vs. AV18.

**Table 3:** RT-PCR Forward/Reverse (F/R) Primers Sequences.

Forward/Reverse	Sequence (5′-3′)
BNP	F: 5′-GACAAGAGAGAGCAGGACACCAT-3′
	R: 5′-TAAGGAAAAGCAGGAGCAGAATCAT-3′
TGF-β	F: 5′-CTAATGGTGGACCGCAACAAC-3′
	R: 5′-CACTGCTTCCCGAATGTCTGA -3′
TNF-а	F: 5′-AGCAAACCACCAAGCGGAGG-3′
	R: 5′-CAGCCTTGTCCCTTGAAGAGAAC-3′
MCP-1	F: 5′-TCTGTGCTGACCCCAATAAGGAA-3′
	R:5′-GAGGTGGTTGTGGAAAAGAGAGTG-3′
Cys-C	F: 5′-CCACCAGGAGACAGTAAAGAAGC-3′
	R: 5′-ATTGAGCAAGAGCAAGGTATGAC-3′
NGAL	F: 5′-GGCTGTCGCTACTGGATCAGA-3′
	R: 5′-GCTTGGTGGAATCATGGCTGG-3′
а-SMA	F: 5′-CTCCCAGCACCATGAAGATCAA-3′
	R: 5′-GGGCGTGACTTAGAAGCATTTG-3′
CRP	F: 5′-AAGCCTTCACTGTGTGTCTCTATGC-3′
	R: 5′-TTCAGGCCCACCTACTGCAATA-3′
IL-6	F: 5′-AGTTGCCTTCTTGGGACTGATGT-3′
	R: 5′-GGTCTGTTGTGGGTGGTATCCTC-3′
IL-1β	F: 5′-ATGTATGCCTACTCATCGGGA-3′
	R: 5′-CAACACAGGCTTGTCTTCTCC-3′
GAPDH	F: 5′-CAACGGGAAACCCATCACCA-3′
	R: 5′-ACGCCAGTAGACTCCACGACAT-3′

BNP, brain natriuretic peptide; TGF-β, transforming growth factor-β; TNF-α, tumor necrosis factor-α; MCP-1, monocyte chemotactic protein 1; Cys-c, Cystatin C; NGAL, neutrophil gelatinaseassociated lipocalin; α-SMA, α-smooth muscle actin; CRP, C-reaction protein; IL-6, interleukin-6; IL-1β, interleukin-1β; GAPDH, glyceraldehydes-3-phosphate dehydrogenase.
